# A Computerized Adaptive Test for the Knowledge of Effective Parenting Test–Internalizing Module: Instrument Validation Study

**DOI:** 10.2196/81646

**Published:** 2026-02-13

**Authors:** Oliver Lindhiem, Hannah D Gallagher, Claire S Tomlinson, Rachel Vaughn-Coaxum, David J Kolko, Paul A Pilkonis, Lan Yu

**Affiliations:** 1 School of Medicine, University of Pittsburgh, 100 N Bellefield, Office 537, Pittsburgh, PA, 15213, United States, 1 412-246-5909; 2 Western Psychiatric Hospital, University of Pittsburgh Medical Center, Pittsburgh, PA, United States

**Keywords:** parenting knowledge, assessment, computerized adaptive testing, internalizing symptoms, psychometrics

## Abstract

**Background:**

The development of efficient, scalable, and precise tools to assess knowledge of evidence-based parenting strategies is critical, particularly as increased parenting knowledge is a core target of many intervention programs.

**Objective:**

This study aimed to develop and evaluate a computerized adaptive testing version of the Knowledge of Effective Parenting Test–Internalizing module (KEPT-I CAT).

**Methods:**

Using computerized adaptive testing simulations from a large (n=1000) national dataset, we compared the performance of the KEPT-I CAT to both the full-length Knowledge of Effective Parenting Test–Internalizing module and a 10-item static short form (KEPT-I Brief).

**Results:**

Results indicated that the KEPT-I CAT achieved comparable efficiency to the KEPT-I Brief (10 items), while demonstrating superior psychometric properties and modestly reducing the potential for practice effects.

**Conclusions:**

Given these advantages, the KEPT-I CAT is well-suited for post-intervention assessment and may facilitate research examining how increases in parenting knowledge relate to changes in behavior and reductions in child internalizing symptoms.

## Introduction

### The Role of Knowledge in Parent Training

Numerous parent training interventions have been developed to address problematic behaviors in children, offering substantial benefits to many families [1]. These interventions train parents in essential skills for behavior management that foster positive changes in their children [2,3]. In measuring baseline parenting and post-treatment outcomes, researchers typically rely on validated assessments of parenting behaviors and practices [4,5]. However, one crucial component that underlies successful behavioral change—parenting knowledge—is often overlooked in studies of program effectiveness [6-9]. Knowledge serves as a foundation upon which skills are built, yet few measures directly and rigorously assess the construct [10]. This limitation hampers accurate assessment of the full impact of interventions (ie, both the knowledge gained and the associations between that knowledge and skill use).

### Knowledge of Effective Parenting Test

The Knowledge of Effective Parenting Test (KEPT) consists of 2 modules designed to assess knowledge of parent management skills [6]. The first module, the 21-item Knowledge of Effective Parenting Test–Externalizing (KEPT-E), assesses parent knowledge related to externalizing behaviors in children. The KEPT-E has demonstrated robust reliability and validity, including expected patterns of converging and diverging correlations between the KEPT and other measures of externalizing child behavior and child and parent psychopathology. The KEPT was recently expanded to include domains of parenting associated with the development of child internalizing symptoms, resulting in the addition of the 22-item KEPT-Internalizing (KEPT-I) module [11]. Item content for the KEPT-I includes parent knowledge of exposure principles, warmth, and behavioral activation, and the measure has demonstrated strong reliability and validity [11]. A 10-item short form of the KEPT-I (KEPT-I Brief) was developed by selecting items with strong psychometric properties and balanced content [11].

Assessing parent knowledge related to internalizing issues is important as internalizing symptoms such as anxiety and depression are among the most common concerns endorsed by parents [12]. Many parent training programs include modules designed to enhance understanding of internalizing symptoms and related parenting strategies [13-15]. Furthermore, evidence-based therapies for child anxiety and depression that include parent education and skill development are among the most efficacious treatments to date [16,17]. However, few measures, other than the KEPT, explicitly assess parent knowledge of these constructs [10,18].

### Computerized Adaptive Testing

Efficient, scalable, and precise assessment tools are necessary for feasible uptake of evidence-based measurement in both research and applied contexts. The full KEPT-I assessment may be less efficient than necessary in certain contexts, owing to the multimodal format that includes video vignettes and results in an average completion time of 25 minutes. Despite its utility, we propose computerized adaptive testing (CAT) as a promising approach to increase the efficiency of the KEPT-I for broader adoption [19,20]. CAT dynamically selects items to adjust question difficulty to the respondent’s ability level using an Item Response Theory (IRT) framework to measure an underlying latent trait typically represented as a standardized score (theta) with a mean of 0.0 and SD of 1.0. In the context of the KEPT, a theta score of 0.0 indicates average parenting knowledge, whereas theta scores of 1.0 and −1.0 represent knowledge scores that are 1 SD above and below the mean, respectively [21,22]. This adaptive model can lead to greater efficiency and shorter test lengths compared with fixed-length counterparts [23,24]. Because CAT selects items from comprehensive item banks, it can administer different items at optimal times depending on a respondent’s current level of a latent trait (or theta), and this feature has the potential to reduce practice effects for respondents. For researchers, use of CAT can lead to improved data quality, shorter administration time, and greater sensitivity to change. Several studies have demonstrated the validity of CAT by showing reductions in measurement error and strong correlations with traditional static measures [24-26].

Within the field of mental and behavioral health, research has consistently demonstrated the effectiveness of CAT in the study of depression [20,24,27]. CAT instruments have also been created for other mental health conditions, including anxiety [28,29], bipolar disorder [30], and psychotic disorders [31,32]. Recognizing the benefits of adaptive testing, a CAT version of the KEPT-E was developed in 2020 and was shown to increase efficiency while maintaining strong psychometric properties [33].

### This Study

In this study, we developed a CAT prototype for the KEPT-I module (KEPT-I CAT) and evaluated its performance relative to the static 10-item short form (KEPT-I Brief) using simulations from the calibration sample of 1000 parents or guardians of children aged 5 to 12 years. The Firestar R package (version 1.9.2; R Foundation for Statistical Computing [34]) was used to conduct simulations. The goal was to find the stopping rules that best balanced the number of items administered and the accuracy of the measure. The development of such a tool holds the potential to enhance the accuracy and efficiency of assessments, thereby contributing to the advancement of research in the field of parenting interventions.

## Methods

### Overview

For the development and evaluation of the KEPT-I CAT prototype, simulations were conducted using the KEPT-I calibration data that were previously collected from a large national sample (n=1000) of parents or guardians (n=567, 56.7% female; mean age 40, SD 7.4 years) of children aged 5 to 12 years. The sample was demographically representative of the US population for key variables including race, ethnicity, income, education, and marital status (n=762, 76.2% White; n=179, 17.9% Hispanic, Spanish, or Latin origin; n=604, 60.4% earning less than US $100,000 annually; n=704, 70.4% with some college education or higher [11]). The KEPT-I demonstrated good internal consistency (α=.82 for the full 22-item version and α=.68 for the 10-item brief version) and showed significant correlations in the expected directions with parenting behaviors, child and parent psychopathology, and mental health service use, supporting its reliability and convergent validity.

**Figure 1.**
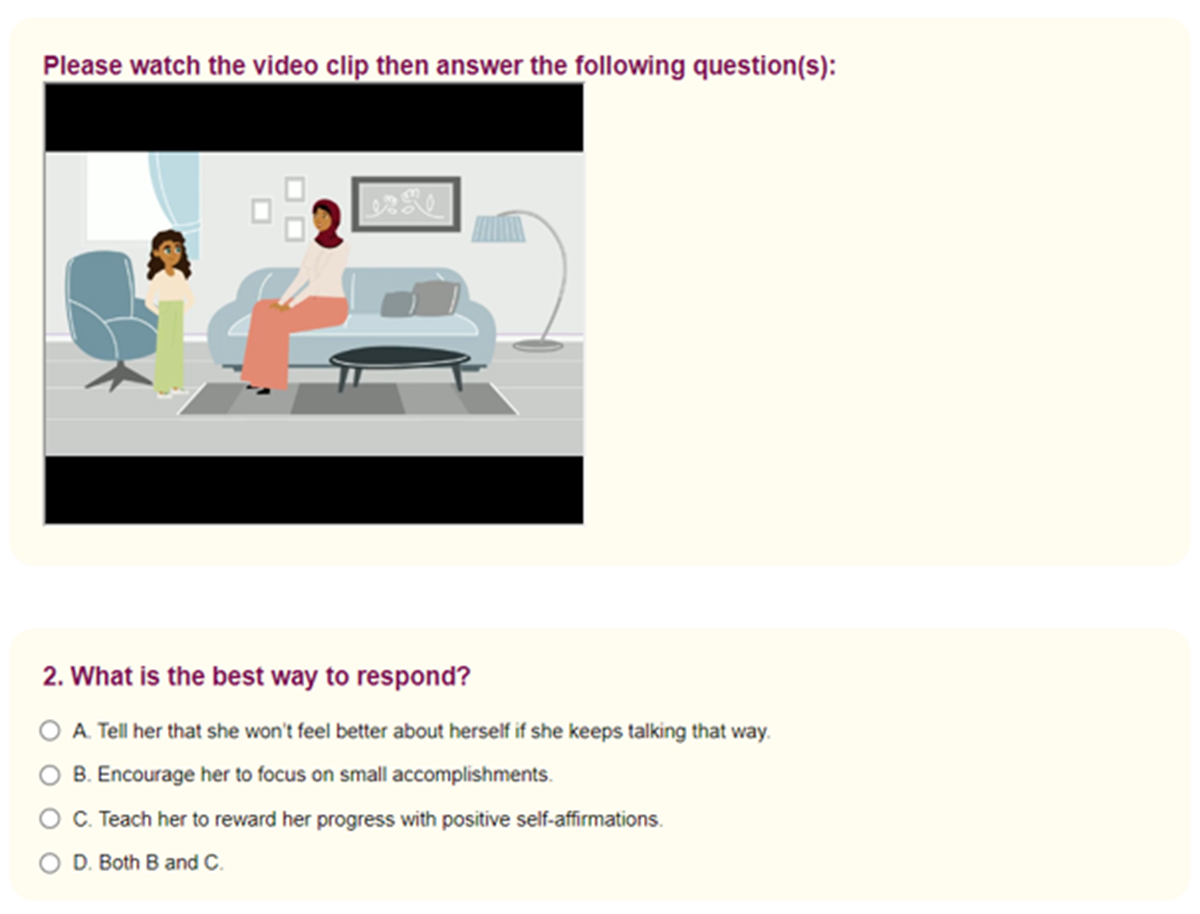
Sample screenshot and item from the Knowledge of Effective Parenting Test–Internalizing module.

A full description of the demographic characteristics of participants and the psychometric properties of the KEPT-I (ie, reliability and validity) is provided elsewhere [11]. The item bank for the KEPT-I CAT prototype included all 22 multiple-choice items from the IRT-calibrated version of the KEPT-I module [11] assessing knowledge of effective internalizing parenting skills across these content domains: exposure principles or encouraging bravery (3 items), relaxation (4 items), warmth (3 items), involvement (1 item), behavior activation (2 items), reducing criticism (5 items), and cognitive reframing (5 items). The multiple-choice items have 4 response options, with only 1 “correct or best” answer. All items are scored dichotomously as correct or incorrect. Figure 1 shows a sample item.

### Ethical Considerations

The study was approved by the Institutional Review Board at the University of Pittsburgh (STUDY23100020), which waived the requirement for written informed consent due to the survey-based nature of the data used for this study. Participants were compensated with a US $25 gift card for completing the full survey. For participant privacy and confidentiality, data were deidentified before analysis as a protective measure to safeguard personal information.

### IRT Calibration

IRT calibration of the items in the full bank KEPT-I was completed before this study, with results reported elsewhere [11]. The original item pool for the KEPT-I included 40 items. Of those, 18 (45%) items were removed due to poor factor loadings, low item discrimination parameter estimates, local dependency, or significant differential item functioning across racial or ethnic groups. The resulting 22-item bank for the KEPT-I CAT prototype includes only items with acceptable discrimination that functioned equivalently for racially or ethnically diverse respondents.

### CAT Simulation Configuration

The Firestar R package, a CAT simulation program (version 1.9.2) [34], was used to conduct post hoc simulations. To perform the CAT simulations, item parameters from the full 22-item bank were entered into Firestar based on the 3-parameter logistic IRT calibration, along with the actual item responses from all 1000 participants in the calibration dataset. Firestar selects items from the full bank to administer one at a time based on one of several user-selected methods. Among the commonly used item selection approaches, maximum posterior weighted information was chosen based on prior evidence that it provides the greatest measurement precision [35]. The maximum posterior weighted information approach uses the information function weighted by the posterior distribution of trait (latent variable) values. The following additional specifications were selected to configure the CAT simulations: the maximum number of response categories across all items was set to 2, the theta range was set as −4.0 to 4.0, the posterior distribution was set as a normal distribution (mean 0.0, SD 1.0), interim theta and full-length theta values were estimated using an expected a priori method, the SE estimation method was set as the posterior SD, the first item location was set at theta 0.0, and the item selection method was set as the prior mean.

### Manipulated Factors

On the basis of the CAT configuration specifications, we evaluated CAT performance by varying 2 CAT stopping rules. The primary stopping rule was termination after SE reached a minimum value. The secondary stopping rule was termination after a maximum number of items was administered. To identify the most efficient stopping paradigm, we crossed the 2 stopping rules in a 2×3 design, that is, the minimum SE was set as 0.50 (to be consistent with the minimum SE used for the KEPT-E CAT) or 0.45 (to examine the impact of requiring additional precision), and the maximum number of items to be administered was set to 15, 18, or 22 (full length).

### Evaluation Criteria

To evaluate CAT performance in comparison with the 10-item static KEPT-I short form (KEPT-I Brief), simulated CAT theta scores were correlated with full-length theta scores. The correlations were then compared with the previously reported correlation between the full-length KEPT-I and KEPT-I Brief [11]. To evaluate the efficiency of each of the 6 CAT scenarios, the mean and median of CAT item length (the number of items administered) and the final values and distributions of theta scores and SEs were compared. To further evaluate the impact of the combined stopping rules, we investigated the cases that did not reach the target SE after the maximum number of items had been administered by identifying the proportion of cases that would have displayed SE reductions greater than 0.01 if additional items had been administered. The overall goal was to decide on the best CAT scenario based on a balance between efficiency and precision.

## Results

### Test Length and Theta Recovery

A median of 10 items (with a minimum of 6 items) was administered across the 15- (IQR 8-13), 18- (IQR 8-12), and 22- item (IQR 8-13) simulations with an SE of 0.50. The corresponding mean SEs were 0.50 (SD 0.03), 0.50 (SD 0.03), and 0.49 (SD 0.02), respectively. A median of 14 items (with a minimum of 7 items) was administered across the 15- (IQR 12-15), 18- (IQR 12-18), and 22-item (IQR 12-21) simulations with an SE of 0.45. The corresponding mean SEs were 0.47 (SD 0.04), 0.46 (SD 0.04), and 0.46 (SD 0.04), respectively. Table 1 shows the individual item length and final estimation for each of the simulation scenarios. Figures S1 and S2 in Multimedia Appendix 1 provide the frequency distributions of simulated CAT lengths across the 6 scenarios. For the 3 scenarios with the SE of 0.50 (Figure S1 in Multimedia Appendix 1), the percentages of cases with CAT terminated after 11 items or fewer (ie, one more item than the median item length) were 63.4% (634/1000), 62.8% (628/1000), and 62.1% (621/1000) for the 22-, 18-, and 15-item CAT, respectively. For the 3 scenarios with the SE of 0.45 (Figure S2 in Multimedia Appendix 1), the percentages of cases with CAT terminated after 15 items or fewer (ie, one more item than the median item length) were 62.7% (627/1000), 62.7% (627/1000), and 100% (1000/1000) for the 22-, 18-, and 15-item CAT, respectively.

**Table 1.** Item length, final estimation, and theta recovery comparisons across the 6 computerized adaptive testing (CAT) simulation scenarios.

	Item length		Final estimation		Theta recovery, RMSE^a^
	Mean (SD; range)	Median (IQR)	Theta, mean (SD; range)	SE, mean (SD; range)	
SE of 0.50					
22-item CAT	11.65 (4.58; 6 to 22)	10 (8-13)	0.03 (0.87; −2.49 to 1.71)	0.49 (0.02; 0.47 to 0.58)	0.262
18-item CAT	11.12 (3.59; 6 to 18)	10 (8-12)	0.02 (0.86; −2.47 to 1.69)	0.50 (0.03; 0.46 to 0.59)	0.249
15-item CAT	10.70 (2.76; 6 to 15)	10 (8-13)	−0.03 (0.86; −2.32 to 1.67)	0.50 (0.03; 0.46 to 0.67)	0.242
SE of 0.45					
22-item CAT	15.29 (4.57; 7 to 22)	14 (12-21)	0.01 (0.88; −2.34 to 1.71)	0.46 (0.04; 0.42 to 0.60)	0.164
18-item CAT	14.17 (3.15; 7 to 18)	14 (12-18)	−0.01 (0.88; −2.50 to 1.69)	0.46 (0.04; 0.42 to 0.59)	0.175
15-item CAT	13.26 (2.04; 7 to 15)	14 (12-15)	0.00 (0.88; −2.50 to 1.67)	0.47 (0.04; 0.43 to 0.72)	0.196

a RMSE: root mean squared error.

To evaluate the theta recovery of each simulation scenario, we further computed root mean squared error (RMSE) with the full-length theta. As shown in Table 1, for the SE of 0.50, the 15-item and 18-item CAT scenarios yielded similar RMSEs, with a slightly higher RMSE in the 22-item CAT. For the SE of 0.45, the 18-item and 22-item CAT scenarios yielded similar RMSEs, with a slightly higher RMSE in the 15-item CAT.

### Secondary Stopping Rule

As shown in Figure S3 in Multimedia Appendix 1, for the 3 scenarios for the SE of 0.50, out of 1000 cases, there were 148 (14.8%), 123 (12.3%), and 109 (10.9%) cases with CAT terminated based on the secondary stopping rule of a maximum number of items for 22-, 18-, and 15-item CAT, respectively. These cases were in tails of the theta score distributions (eg, for the 123 cases from the 18-item CAT, n=26, 21.1% of the cases with theta below −1.0% and n=95, 77.2% of the cases with theta above 1.0, with a mean SE of 0.55 and a range of 0.50-0.59). For the 3 scenarios for the SE of 0.45 in Figure S4 in Multimedia Appendix 1, there were 247 (24.7%) cases, 282 (28.2%) cases, and 384 (38.4%) cases with CAT terminated based on the secondary stopping rule of a maximum number of items for the 22-, 18-, and 15-item CAT, respectively. Most of these cases were again in the tails of the theta score distributions (eg, for the 282 cases from the 18-item CAT, n=97, 34.4% of the cases with theta below −1.0% and n=130, 46.1% cases with theta above 1.0, with a mean SE of 0.51 and a range of 0.45-0.59). These results are consistent with the fact that IRT-calibrated tests typically display less precision in the tails of test information distributions, where there is less variability in responses, that is, when respondents are providing all correct (or incorrect) answers, each individual item becomes less discriminating.

### Comparison With the Full-Length Test

**Figure 2.**
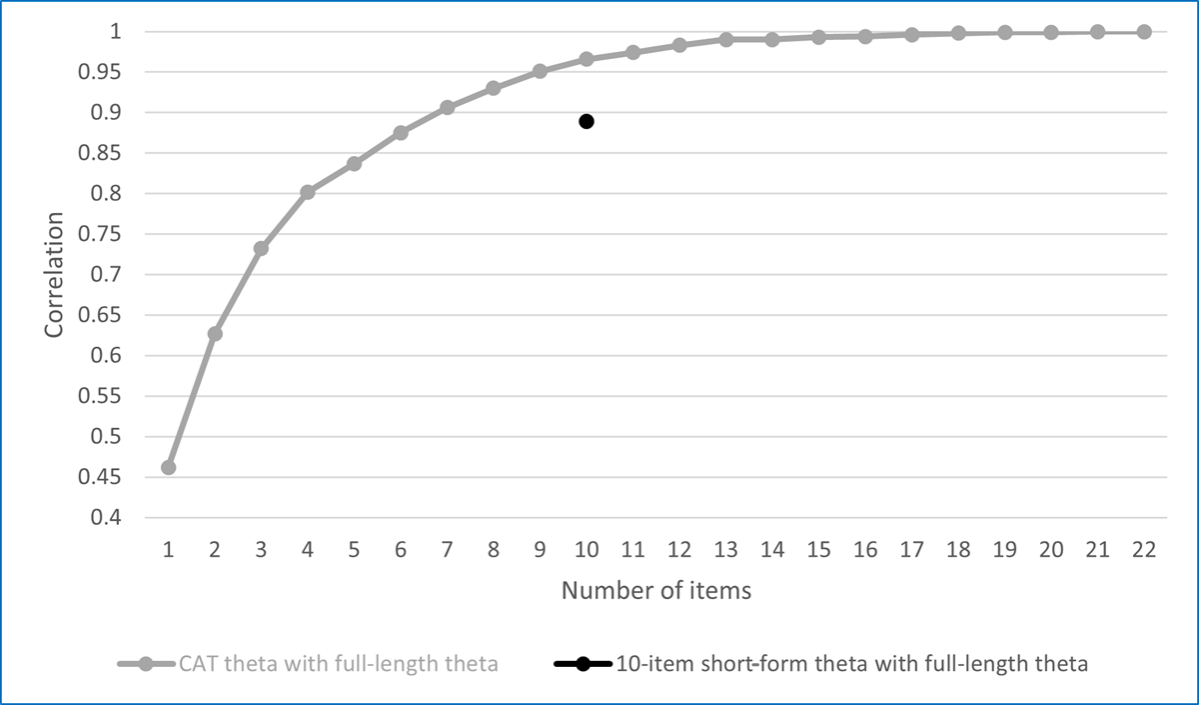
Correlations between computerized adaptive testing (CAT) and static short-form thetas and full-length thetas for the SE of 0.50.

**Figure 3.**
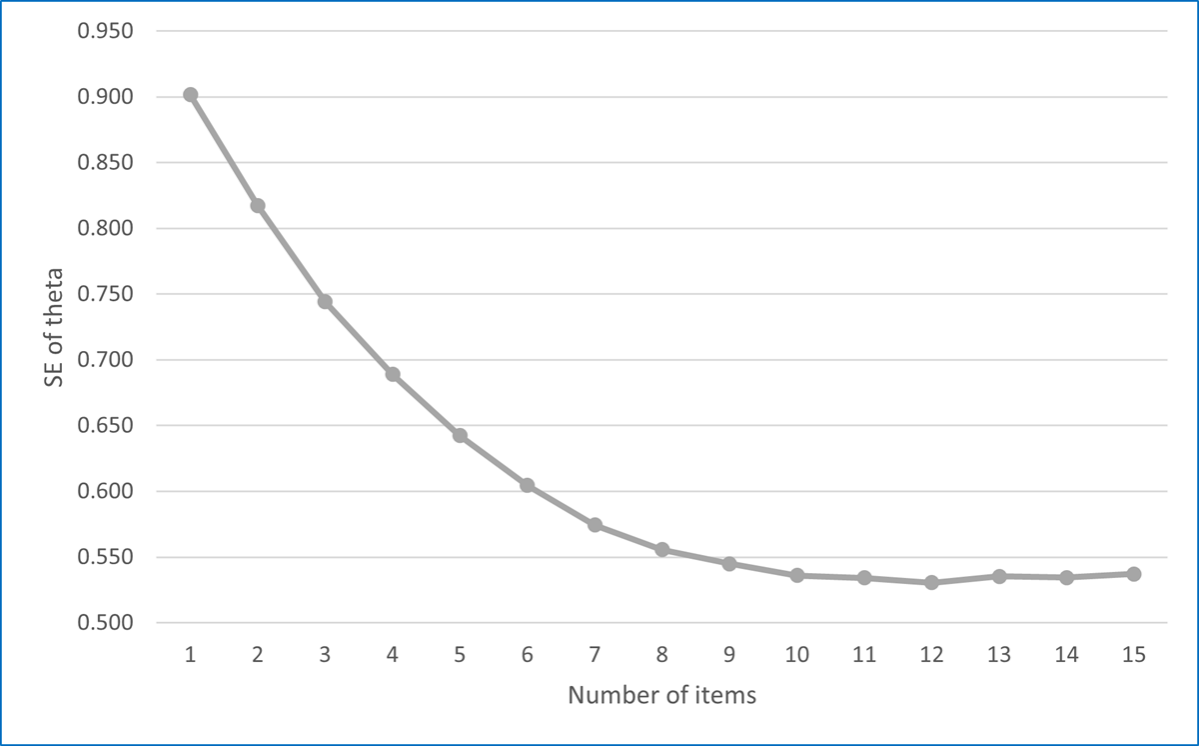
SE change over the 15-Item computerized adaptive testing with the SE of 0.50.

The correlations between 9-item CAT theta and full-length theta reached 0.95 for both the SE of 0.50 and 0.45 scenarios, which exceeds the correlation between the KEPT-I Brief and the full form (*r*=0.90). Figure 2 summarizes the correlations between CAT theta scores varying by length and the full-length theta for the SE of 0.50 model. The correlation between CAT theta and full-length theta exceeds 0.90 with as few as 7 items. The figure also shows the correlation between the 10-item (short) static form and the full static form. On average, the 10-item static form offers the same precision as between 6 and 7 CAT-sequenced items, highlighting the benefit of CAT sequencing. Figure 3 shows the distribution of SEs of theta estimates as a function of test length for the 15-item CAT with the SE of 0.50.

### Impact of Additional Items on SE Reduction

**Figure 4.**
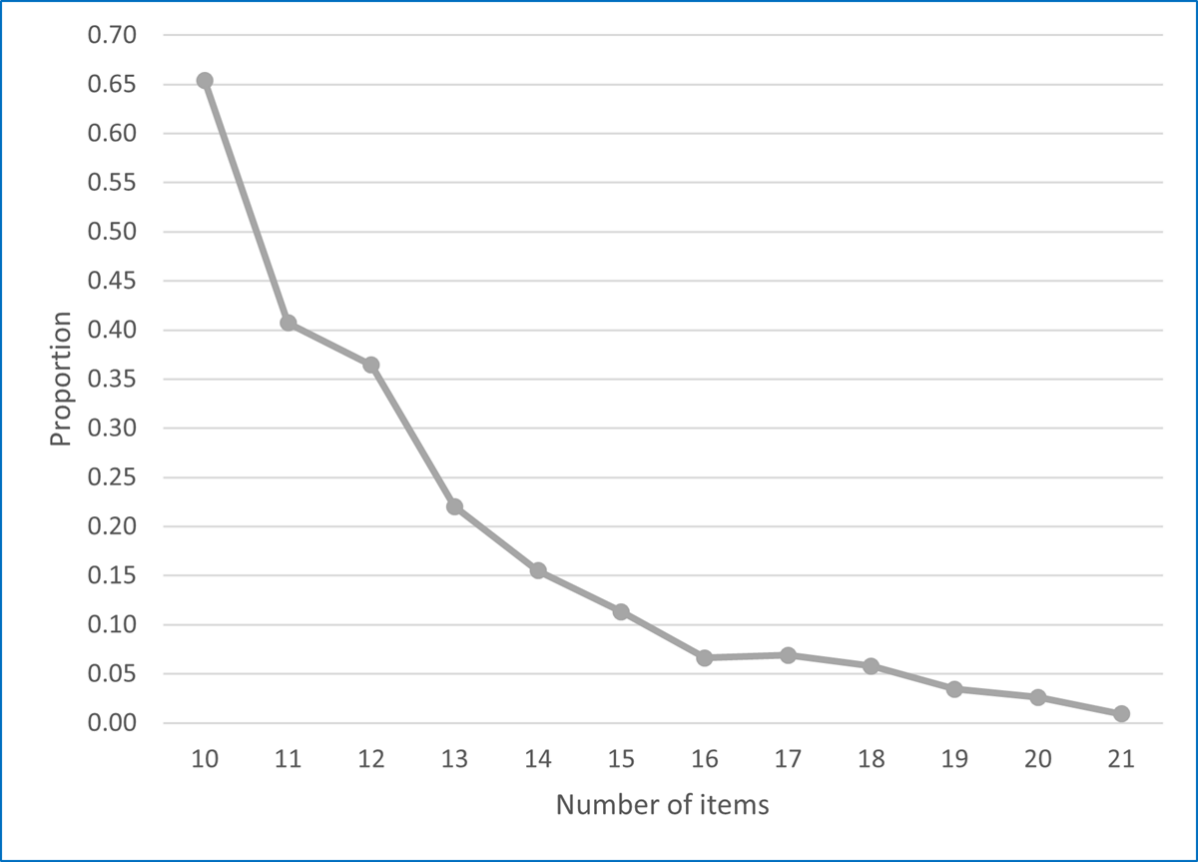
Proportion of SE >0.01 for participants taking the next item.

Figure 4 summarizes the proportion of respondents with a SE reduction >0.01 for each next item between 10 and 21 items. Of the 471 cases that needed to take an additional item after completing 10 items, 308 (65.4%) had SE reductions larger than 0.01. The percentage of cases with an SE reduction greater than 0.01 decreased to 11.3% (20/177) among participants who required an additional item after completing 15 items. Additional items to the 16-item CAT only gained 6.6% (11/166) of cases with a SE reduction larger than 0.01. Again, because of these modest gains in SE reduction, when comparing stopping rules with the SE of 0.50 and SE of 0.45, the median number of items to be administered increased from 10 to 14. In summary, based on the preferred balance between efficiency and precision, the 15-item CAT with the SE of 0.50 was the best selection among the 6 CAT scenarios. Figures4  5 provide the final theta distribution and item frequencies for the 15-item CAT with the SE of 0.50 (Multimedia Appendix 2).

**Figure 5.**
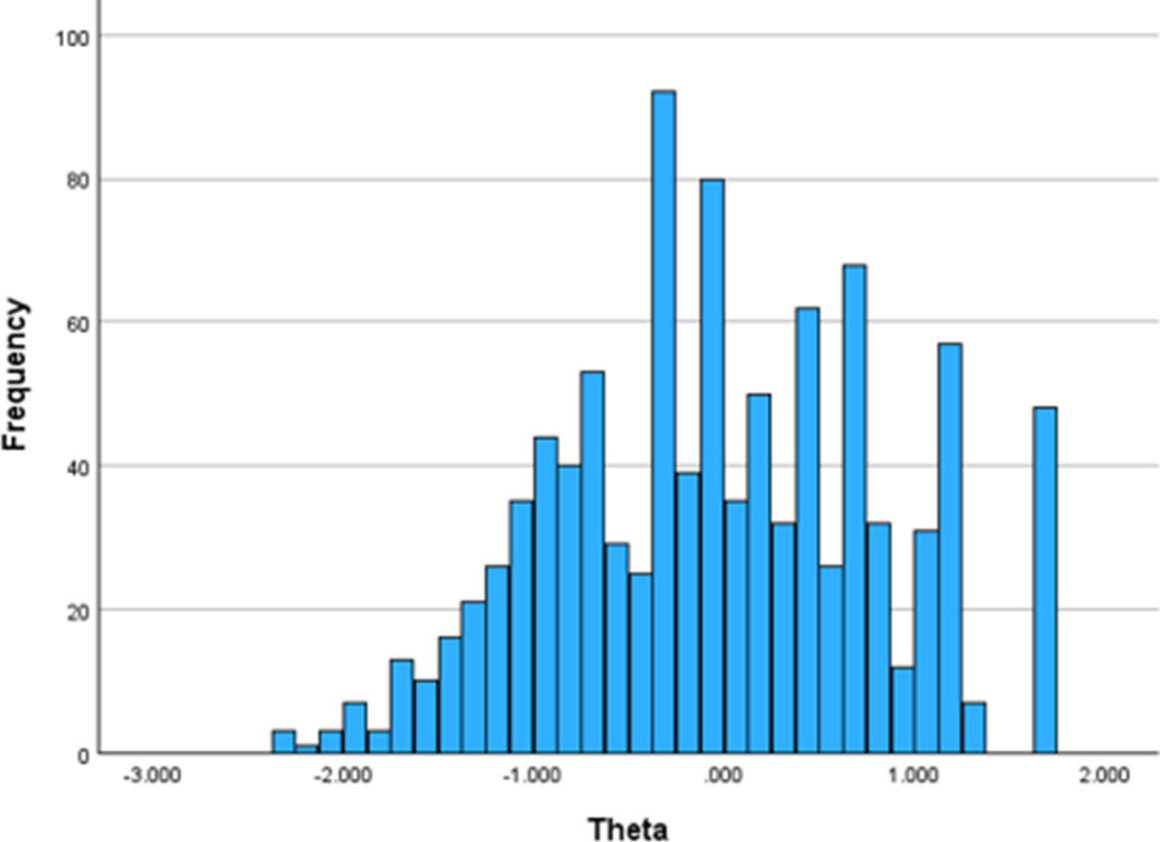
Final theta score distribution of the 15-item computerized adaptive testing with the SE of 0.50 (N=1000; mean –0.031, SD 0.862).

## Discussion

### Principal Findings

In this study, we developed a CAT version of the KEPT-I (KEPT-I CAT), which maintained the strong psychometric properties of the original measure while improving efficiency (albeit modestly). Although the KEPT-I CAT prototype is similar in length to the static 10-item KEPT-I Brief, the adaptive format tailors item selection to each respondent’s latent level of parenting knowledge (ie, IRT ability), thereby maximizing information at each administration. Consequently, the KEPT-I CAT demonstrates incrementally stronger correlations with the full-length KEPT-I than the brief form, indicating greater fidelity to the original measure. These findings suggest that the KEPT-I CAT offers a marginal advantage over the static brief form by achieving comparable efficiency while providing more accurate and reliable estimates of overall parenting knowledge, particularly in research and clinical contexts where precision is prioritized. Another potential advantage of the KEPT-I CAT is its ability to modestly mitigate practice effects. Although the KEPT-I CAT is similar in length to the KEPT-I Brief, adaptive item selection may reduce repeated exposure to identical items across administrations. Given the limited item pool, any benefit in this regard is likely to be incremental.

The development of the KEPT-I CAT contributes to the emerging applications of adaptive testing in mental health research. While CAT has been used in studies of various mental health conditions [20,24,27], the CAT adaptation of the KEPT-I is the first to assess domains of parenting knowledge shown to be associated with the development and maintenance of child internalizing disorders [36-38]. While parenting involvement and knowledge are common targets of child internalizing disorder interventions [2,39], the KEPT-I is one of the few measures with strong psychometric properties designed to assess knowledge of parenting skills related to internalizing disorders [11]. The brevity and process of the KEPT-I CAT make it particularly useful for inclusion in lengthy test batteries, where participants may experience considerable assessment burden and where practice effects may occur if the static KEPT-I Brief is used.

### Strengths and Limitations

The strengths of this project include a large national dataset used to evaluate the performance of the full-length, brief, and CAT versions of the KEPT-I module. The 22 items of the full KEPT-I module that were used as the item bank for the CAT version were rigorously selected from 40 original items using factor analysis and IRT analyses. High factor loadings, strong item parameters, and robust test information curves supported the reliability of all items. In addition, all items were free from significant differential item functioning by race or ethnicity [11].

In terms of limitations, because the item pool has a test information function with greater precision in the middle of the latent trait distribution than in the tails (as is typical of IRT-calibrated tests), measurement precision was more difficult to achieve for respondents in the tails of the distribution. Consequently, a secondary stopping rule was implemented to prevent the KEPT-I CAT from cycling through all the items in the item pool. In addition, because of the relatively small item pool, the CAT prototype does not include content balancing across all domains of parenting knowledge regarding internalizing problems, a feature sometimes used in longer adaptive tests to ensure equal coverage of items from all content areas.

### Summary, Conclusions, and Future Directions

While CAT assessments are valuable tools for streamlining screening and assessment, it is imperative that these tools are scrutinized methodically for reliability and validity according to their intended use, as their utility may vary depending on the context [39]. With strong psychometric properties and improved efficiency, the KEPT-I CAT has the potential to serve as a valuable supplement to measures that directly assess parenting behaviors. The KEPT-I CAT’s capacity to assess parenting knowledge will aid in further exploration to determine how parenting skills knowledge translates to skill use and how this could improve effective parenting strategies to reduce child internalizing symptoms. Further research is needed to validate the KEPT-I CAT in applied settings, including comparisons of its precision with the full 22-item KEPT-I in independent samples. Additional validation in clinical populations will also be important for examining real-world utility.

## Supplementary material

10.2196/81646 Multimedia Appendix 1 Test length and theta distributions for all models tested.

10.2196/81646 Multimedia Appendix 2 Number of items used in 15-item computerized adaptive testing (SE 0.50).

## Abbreviations

CAT: computerized adaptive testing
IRT: item response theory
KEPT: Knowledge of Effective Parenting Test
KEPT-E: Knowledge of Effective Parenting Test–Externalizing
KEPT-I: Knowledge of Effective Parenting Test–Internalizing
KEPT-I Brief: 10-item short form of Knowledge of Effective Parenting Test–Internalizing
KEPT-I CAT: Knowledge of Effective Parenting Test–Internalizing computerized adaptive testing
RMSE: root mean squared error
