# Supplementary material for: A Computerized Adaptive Test for the Knowledge of Effective Parenting Test–Internalizing Module: Instrument Validation Study
Source: JMIR Form Res. 2026 Feb 13;10:e81646. doi: 10.2196/81646 (PMC12904350; doi:10.2196/81646)

*Supplemental Figure 1*. Item length frequencies for SE = 0.50.


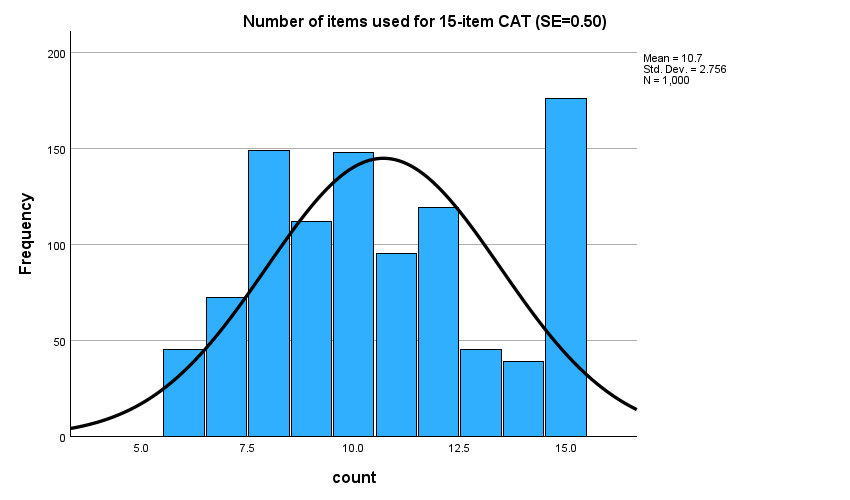


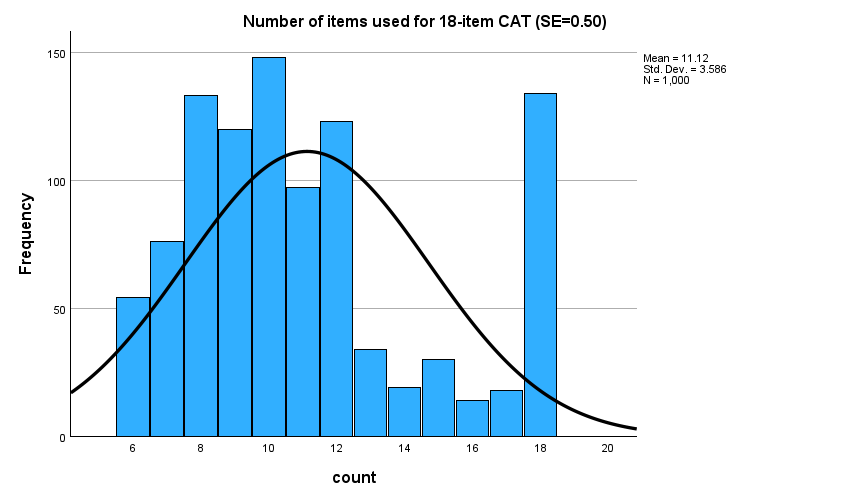


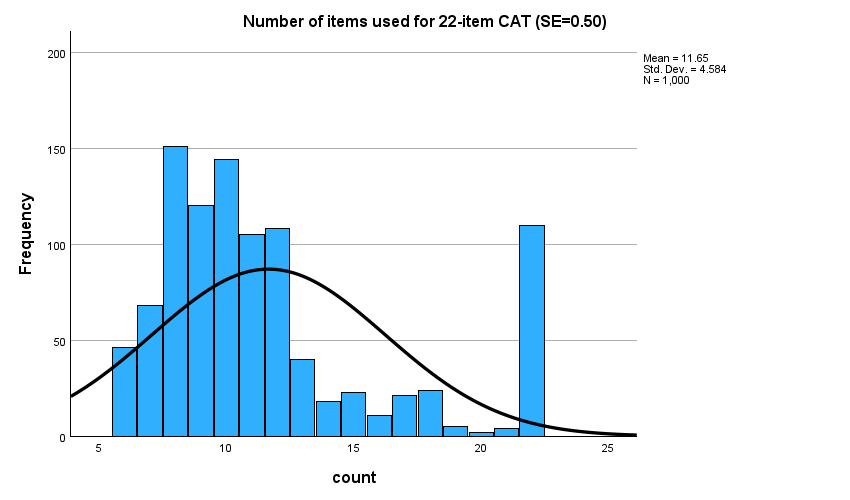


*Supplemental Figure 2*. Item length frequencies for SE = 0.45.


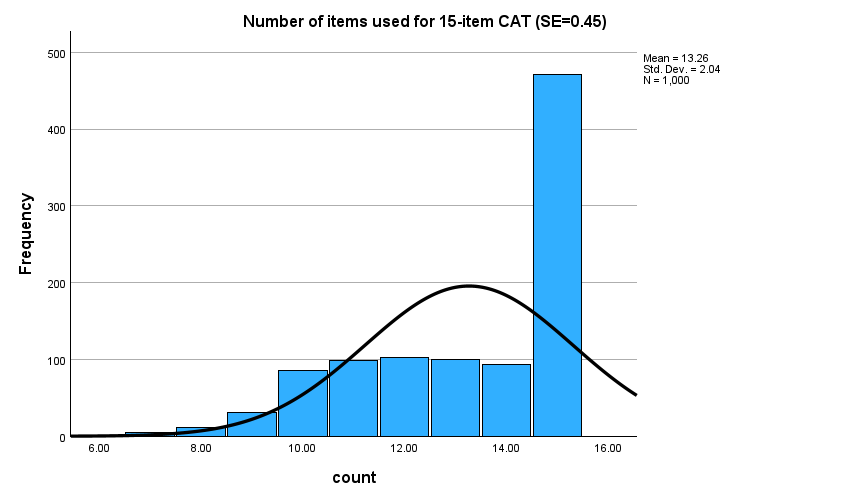


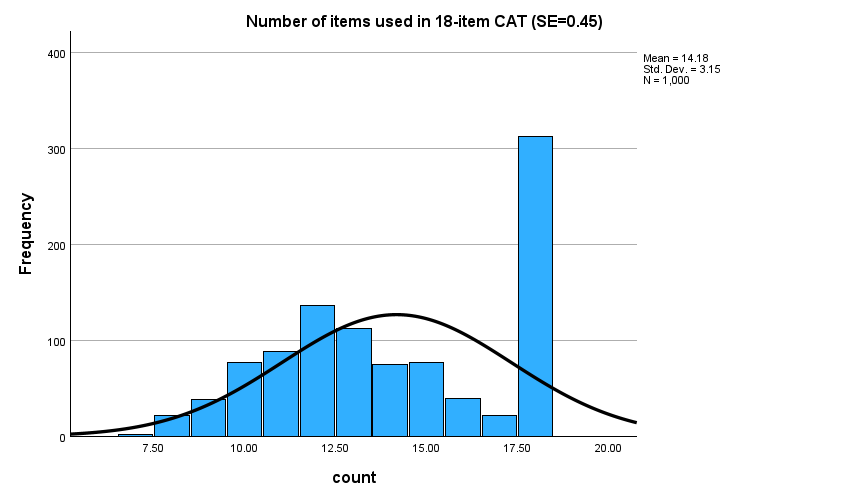


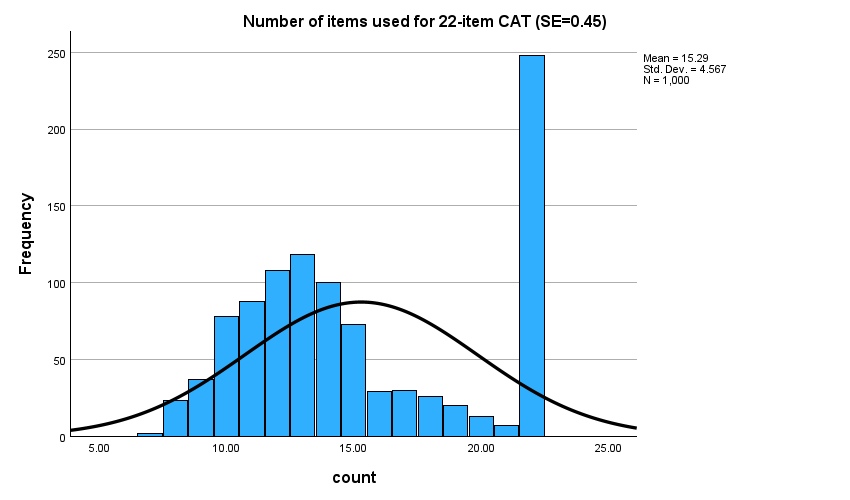


*Supplemental Figure 3*. Theta distributions of the cases with final SE > 0.50.

*
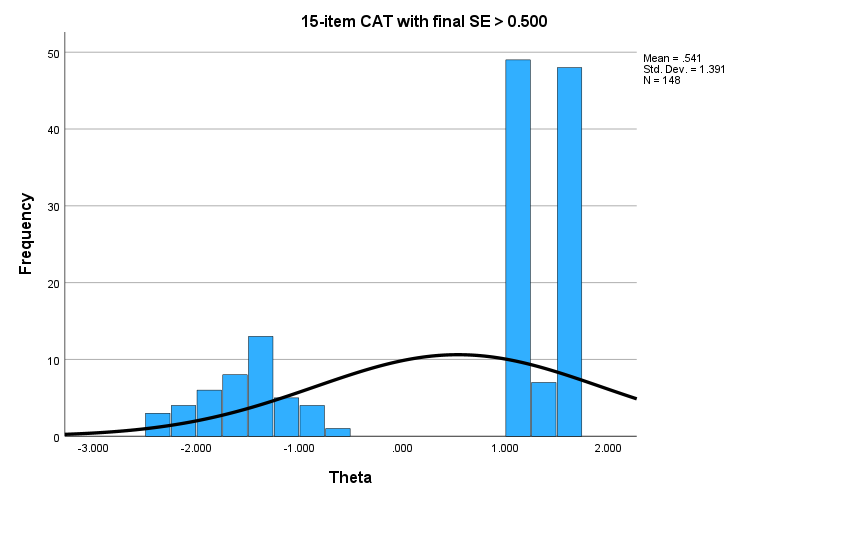
*


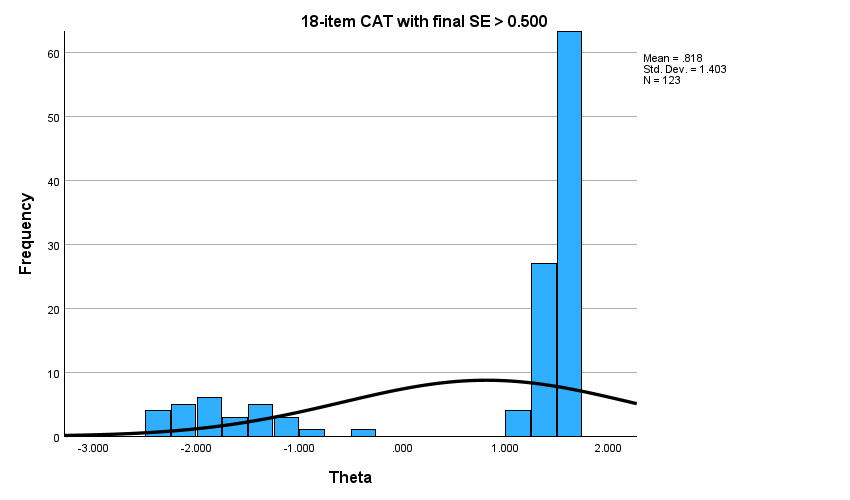


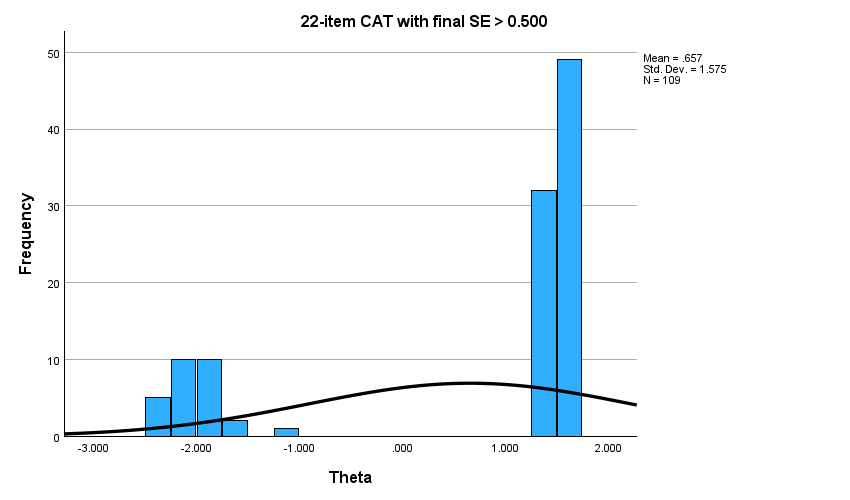


*Supplemental Figure 4*. Theta distributions of the cases with final SE > 0.45.


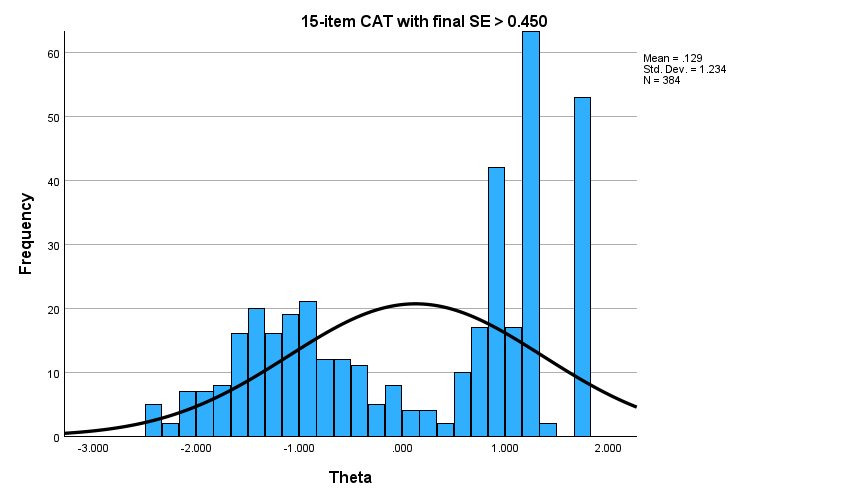


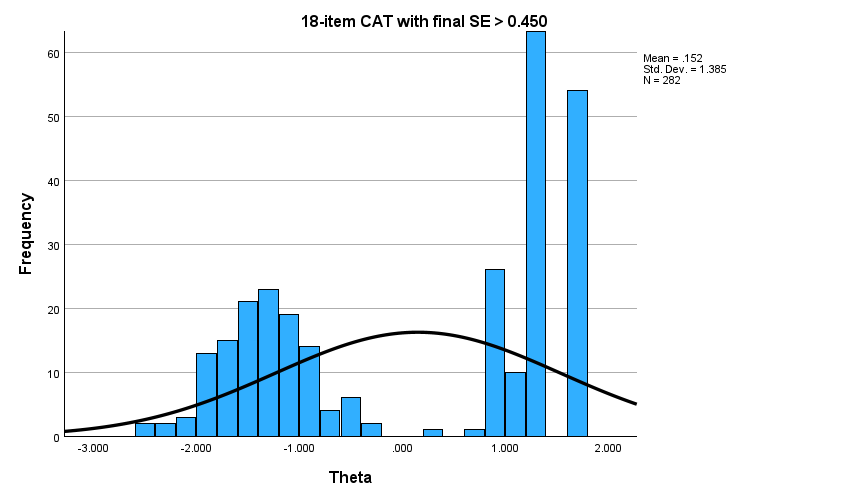


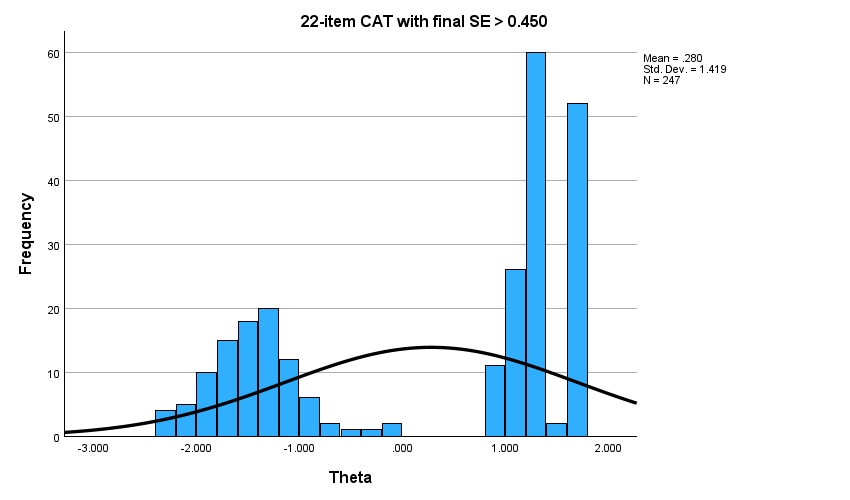

Supplement: Multimedia Appendix 1 [file formative-v10-e81646-s001.docx]
