# Supplementary figures and images for: A Computerized Adaptive Test for the Knowledge of Effective Parenting Test–Internalizing Module: Instrument Validation Study
Source: JMIR Form Res. 2026 Feb 13;10:e81646. doi: 10.2196/81646 (PMC12904350; doi:10.2196/81646)

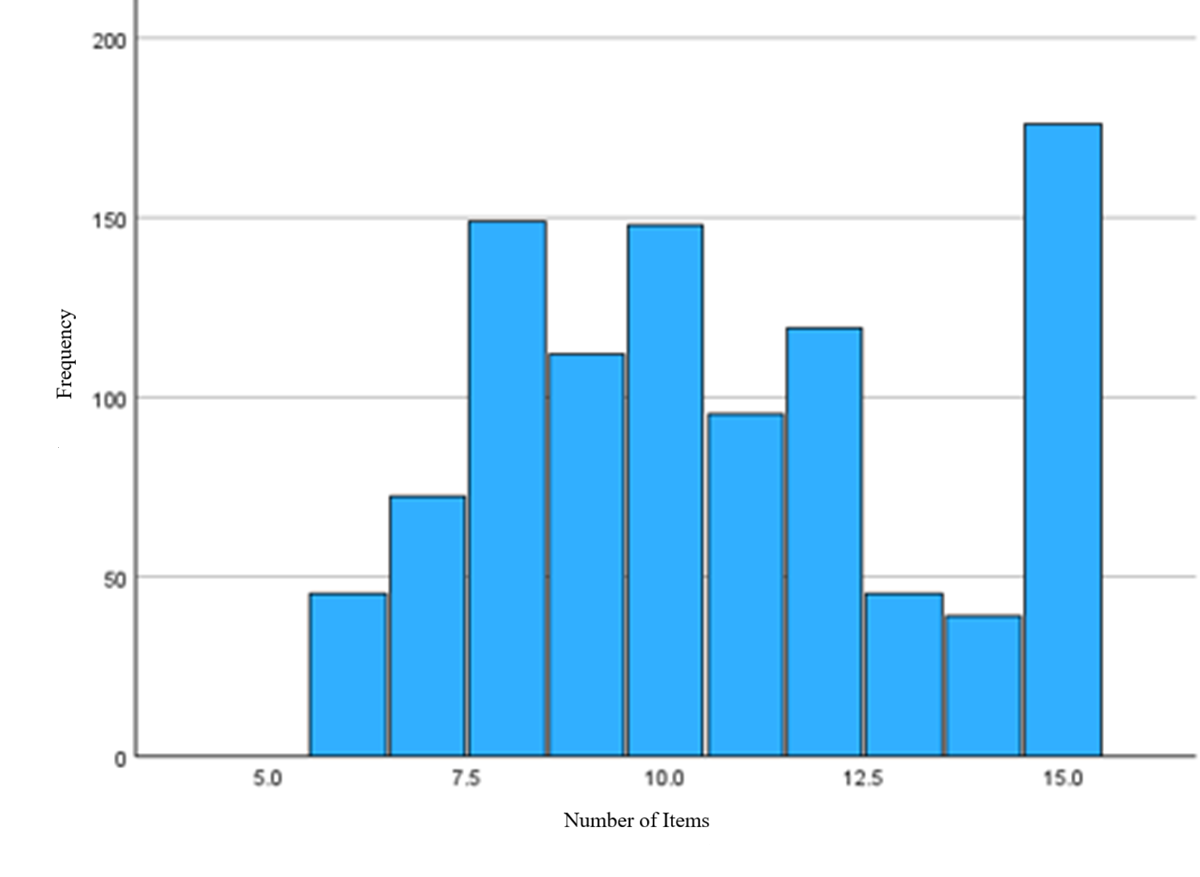

Supplement: Multimedia Appendix 2 [file formative-v10-e81646-s002.png]
